# Caries progression in non-cavitated fissures after infiltrant application: a 3-year follow-up of a randomized controlled clinical trial

**DOI:** 10.1590/1678-7757-2016-0633

**Published:** 2017

**Authors:** Camillo ANAUATE-NETTO, Laurindo BORELLI, Ricardo AMORE, Vinicius DI HIPÓLITO, Paulo Henrique Perlatti D’ALPINO

**Affiliations:** 1 Universidade Metropolitana de Santos, Centro de Pós-Graduação, Santos, SP, Brasil.; 2 Clínica particular, São Paulo, SP, Brasil.; 3 Universidade Mogi das Cruzes, Curso de Odontologia, Mogi das Cruzes, SP, Brasil.; 4 Universidade Anhanguera de São Paulo, Pós-Graduação Stricto Sensu em Biotecnologia e Inovação em Saúde, São Paulo, SP, Brasil.

**Keywords:** Clinical trial, Dental enamel, Pit and fissure sealants, Operative dentistry, Oral diagnosis, Dental radiography

## Abstract

**Objectives:**

To evaluate the efficacy of a conservative treatment to prevent the progression of caries using an infiltrant on non-cavitated pit and fissures.

**Material and Methods:**

This controlled clinical trial selected 23 volunteers with clinically and radiographically non-cavitated occlusal caries among patients presenting a “rather low” to “very high” caries risk. Eighty-six teeth were randomly divided into two experimental groups: teeth receiving a commercial pit-and-fissure sealant (Alpha Seal-DFL) and contralateral teeth receiving Icon infiltrant (DMG). Caries progression was monitored by clinical (laser fluorescence caries detection) and radiographic examination at 12-month intervals over a period of 3 years of monitored caries progression. Probing the sealing materials to detect areas of retention was also used to evaluate marginal integrity.

**Results:**

Statistical analysis showed no difference in caries progression using laser fluorescence caries detection when both materials were compared, regardless of the evaluation times (p>0.05). No significance was observed when the marginal sealant integrity of both materials was compared, regardless of the evaluation time (p<0.05). Marginal integrity significantly reduced after 1 year for both materials (p<0.05), but remained stable after 2 and 3 years of evaluation, compared with 1-year results (p>0.05). SEM analysis exhibited a more homogeneous sealing for the infiltrant than obtained by the sealant.

**Conclusions:**

The infiltrant was effective to prevent the caries progression in non-cavitated pit-and-fissures after 3 years of clinical evaluation, comparable with the conventional sealant. The infiltrant also presented better results in terms of caries progression at the 3-year evaluation time using the radiographic analysis.

## Introduction

The configuration of pits and fissures is clinically relevant to determine the susceptibility for occlusal caries when the factors are present^29^. Sealants prevent caries by reducing available retention sites forming a smooth surface layer and providing the inhibition of bacterial survival by preventing nutrients from reaching microflora in the fissures^29^. Therefore, differently from glass ionomer sealants that can be lost maintaining their anti-cariogenic effect, the clinical effectiveness and success of resin sealants have been related with their retention^30^. If the sealant is fully retained, then possibly recurrent caries or progression of caries beneath the restoration is negligible^19^. To enhance the longevity of pit-and-fissure sealants, several materials and techniques have been developed^11^.

Caries lesions are characterized by demineralization in the lesion body, whereas the surface remains comparably highly mineralized^18^. In an early stage, these lesions can be arrested or even remineralized^9^. Conventional fissure sealing results only in a superficial resin penetration on the pit and fissure, establishing a preventive mechanical barrier. Therefore, its indication relies on sound and/or slight demineralized superficial surface, which is clinically difficult to define. In this scenario, low-viscosity infiltrant represents a promising alternative approach. A promising alternative therapy to arrest caries lesions relies on the infiltration of low-viscosity, photoactivated resins in their subsurface^17^. The application of an infiltrant resin has been claimed to improve caries infiltration^17^. It erodes the pseudo-intact and relatively impermeable surface layer with hydrochloric acid gel, desiccating the lesion with ethanol. Then, a subsequent application of the infiltrant material takes place^24^. In contrast to caries sealing, caries infiltration removes any excessive resin on the lesion surface before the material is light cured^21^. Consequently, caries inhibition comes mostly from occlusion of the pores within the lesion body, which slows down diffusion^27^.

Resin infiltration seems to result in considerably deeper resin penetration whereas pre-treatment with hydrochloric acid seems more suitable compared with the application of phosphoric acid^21^. Regarding clinical practice, this modified etching technique is claimed to reduce the influence of the highly mineralized surface layer on infiltration abilities into fissure caries lesions^21^. In addition, the ability of the infiltrant to effectively infiltrate the enamel lesions may allow better clinical results^23^. Enamel caries present pores that may act as diffusion pathways for acids and dissolved minerals. Therefore, occluding these pores with photoactivated resins might arrest the progress of the caries lesion and mechanically stabilize the structurally fragile lesion^23^. A systematic review of *in vivo* studies revealed that resin infiltration seems to be an effective method to arrest the progression of non-cavitated proximal caries lesions extended radiographically at maximum to the outer third of dentin in combination with non-operative measures compared to non-operative measures applied alone^6^.

The rationale that support the use of infiltrant as a sealing material relies on fact that, although the protocol for the application of the former is completely different from that of a conventional sealant, it is not possible to obtain a shallow layer of the infiltrant when applied in occlusal pit-and fissures. The manufacturer’s specifications for the resin infiltration technique comprise two steps: first, after erosion of the pseudo-intact surface layer, the infiltrant with low viscosity penetrate the body of lesion driven by capillary forces. In this way, porosities of the carious lesion are occluded. Then, there is a second step in which the manufacturer recommends reapplication of Icon-Infiltrant. Thus, the morphological and physical characteristics of the infiltrant resemble the clinical aspect of a conventional sealant after application in occlusal fissures.

Paris, et al.^21^ (2014) evaluated *in vitro* ^21^ the penetration of an infiltrant and a sealant, when applied, as recommended, into fissure caries lesions. These authors justified the use of the infiltrant on the fact that the resin infiltration technique was primarily developed to arrest proximal lesions and that this technique had not been evaluated for fissure caries lesions so far^21^. It was demonstrated that the fissure sealing resulted only in a superficial penetration of the resin. On the other hand, resin infiltration homogeneously filled up about half as much fissures as the sealant^21^. A previous study^17^ also demonstrated that the infiltrant showed superior ability to penetrate natural lesions compared to commercial adhesives *in vitro*. The authors advocated that the effect of a deeper penetration on the inhibition of lesion progression should be evaluated in future studies. In another study^3^, a clinical trial evaluated the efficacy of infiltrating, sealing, or fluoride varnishing on the occlusal surfaces with initial caries lesions in the primary dentition for up to 3 years. Both infiltrant and sealant materials were found to effectively seal initial caries lesions on occlusal surfaces of primary molar teeth sealed, arresting caries progression. However, to date, no clinical trials evaluating the infiltration of non-cavitated fissure lesions in the permanent dentition are available.

This study aimed to clinically evaluate the effectiveness of a conventional pit-and-fissure sealant and a resinous infiltrant used in the same way in non-cavitated fissures. The criteria were their capacity to hamper the progress of caries evaluated by radiographic analysis, laser fluorescence, and the long-term superficial integrity at four levels: baseline, after 1, 2, and 3 years. The research hypothesis tested was that caries prevention and marginal integrity in non-cavitated fissures would be less effective under the application of the infiltrant as a sealing material compared with a conventional sealant.

## Material and methods

### Ethical considerations

The project was approved by the Research Ethics Committee of Universidade Anhanguera de São Paulo, Brazil (protocol 20090103/2009). All patients or their relatives received information about the study and signed a free informed consent form according to Resolution 196/6 of the National Health Council and the Declaration of Helsinki (2000). Before the trial, children and their parents were informed about pit-and-fissure sealing. The parents/guardians signed informed treatment consent forms if they agreed to participate in the trial. The volunteers were trained and motivated to brush and floss during the study.

### Experimental design

This controlled clinical trial followed the CONSORT statement. The sample consisted of 86 superior and/or inferior molars from volunteers that presented intact deep and retentive fissures to visual inspection. A single trained and calibrated operator performed visual and radiographic examinations. The operator performed visual examination after pumice and water prophylaxis, through a flat mirror and triplex syringe and headlight. For the radiographic analysis, bite-wing radiographs were taken using a positioner to evaluate the initial tooth integrity before the application of the sealing materials. A total of 23 patients (15 women and 8 men), who were seeking routine dental care at the local clinics, were selected. The mean age of the patients was 14.4 years, ranging from 8 to 24 years old. The criteria for inclusion were teeth in contact with the antagonist tooth, presenting visual non-cavitated caries lesions located between the enamel-dentin junction and middle one-third of dentin. Exclusion criteria were restorations and cavitations on other tooth surfaces. Teeth reported as sensitive to any type of stimulus were also excluded. Physically and mentally challenged volunteers with systemic diseases under medication and children with poor oral habits affecting occlusion were excluded from the study. The same single trained examiner assessed and graded the fissure system independently, according with the International Caries Detection and Assessment System (ICDAS). Teeth were scored as ICDAS codes 0, 1, 2. Most lesions were classified as ICDAS 2 and some were ICDAS 1 or ICDAS 3. They were randomly allocated to two treatment groups (“conventional sealing” and “resin infiltration”). The cariogram model (Bratthall Cariogram) was assessed to estimate the caries risk.

All volunteers got a split-mouth experimental design and an infiltrant (Icon Infiltrant, lot #624261, DMG Dental Materials, Hamburg, Germany) applied to the occlusal surface of maxillary and mandibular molars, so that a commercial pit-and-fissure sealant sealed the contralateral molars (control group) with non-cavitated carious lesions (Alpha Seal Light, lot #2CU8300, DFL, Rio de Janeiro, RJ, Brazil). Two calibrated operators performed all the clinical steps. Before that, a randomization was set up to determine the teeth (contralateral left or right upper/lower molars) that would receive the sealing materials. The dependent variables studied were presence of cavities using laser fluorescence method, occlusal marginal integrity analysis, and also the presence or absence of clinical and radiographic progression of caries.

### Clinical steps

Baseline impressions used a polyvinyl siloxane-based material (Honigum, DMG Dental Materials, Hamburg, Germany) after the application of the sealing materials. For the radiographic examination, a standardized biting holder and bitewing radiographs certified that carious lesions were not present at baseline. Radiographs were taken using the same X-ray source at 70 kVp and 10 mA, and same exposure time (at 0.63 s). The radiographs were manually processed in developing and fixative solutions. After prophylaxis with pumice and water, clinical examination was performed with a flat mirror, triple syringe, and headlight to evaluate the ability to hamper the progression of caries.

Both techniques of application of the sealing materials were performed using a non-invasive technique. The occlusal surfaces were cleaned using a rotary brush and non-fluoridated polishing paste, thoroughly rinsed with a water spray, and dried with the air syringe. A rubber dam was placed before the application of the materials. Considering that both sealing techniques were applied to contralateral teeth, participants were randomly assigned following simple randomization procedures (computerized random numbers) to 1 of 2 treatment groups. The teeth to which the infiltrant was applied received an application of Icon Etch (15 % hydrochloric acid gel – HCl) for 2 min. Then, the occlusal surfaces were rinsed with water for at least 30 s, and dried. Icon Dry was applied onto the pit and fissures for 30 s, and dried. The infiltrant was applied onto the etched surface and set for 3 min. The fissures were filled with the material up to one third to half of the cusps. The excess material was removed with a microbrush. Finally, a LED light (Bluephase, Ivoclar Vivadent, Schaan, Schaan, Liechtenstein), with a radiant emittance of 1000 mw/cm^2^, light-cured the infiltrant for 40 s. The infiltrant was then reapplied and set for 1 min. The excess material was again removed with a microbrush, and photoactivated for 40 s. The tip was positioned over the teeth on the center of the occlusal surface, thus permitting light irradiation throughout the surface of the infiltrant.

In the same way, the contralateral teeth were submitted to prophylaxis using pumice and a photoactivated pit and fissure sealant (Alpha Seal – DFL), was applied according to the manufacturer’s instructions, as follows: the enamel surface was etched using 37% phosphoric acid gel for 60 s, water-rinsed thoroughly for 10 s, and dried. The material was applied with a sharp explorer to avoid excessive spreading of sealant, and light cured for 20 s using the same light curing device. In the same way, the fissures were filled about one third to half of them. The excess material was also removed using a microbrush. Visual inspection then evaluated both sealant and infiltrant coverages, using a dental probe to detect any pit or fissure region not covered by the material.

After photoactivation with the same light curing unit, the rubber dam was removed. Then, the occlusion was checked with a carbon marker and premature contacts were relieved to ensure that the materials would not produce occlusal interferences. Finally, polishing cups were used for the surface finish. Patients were then advised regarding oral hygiene. Impressions using the same material (Honigum, DMG Dental Materials, Hamburg, Germany) were taken after the application of the materials.

For data organization and comparison, digital photographs of the teeth before and after receiving either infiltrant or sealant were also taken and recorded at all of the experimental evaluation times using a digital camera (D100 digital camera/Medical Nikkor lens 120 mm f/4.0 IF, Nikon Corporation Inc., Tokyo, TY, Japan).

### Caries detection using laser fluorescence method

A laser fluorescence method was used to caries detection (DIAGNOdent Pen, KaVo, Biberach, BW, Germany) in non-cavitated molars in different areas of the occlusal surface, giving values from 0 (no fluorescence) to 99 (maximum fluorescence). A single operator calibrated the equipment prior to each examination using the reference given by the manufacturer. The laser tip emitting a wavelength of 655 nm scanned different surfaces of teeth in contact mode showing the amount of demineralization. Caries detection by laser fluorescence 5 points scoring the peak value (0-99) of the occlusal surface. Scores were rated using four classifications, as follows:

Score 1- from 0 to 13: sound;

Score 2- from 14 to 20: enamel cavity;

Score 3- from 21 to 29: deep enamel cavities;

Score 4- higher than 30: cavity at dentinal level.

Caries detection using fluorescence method was performed before the application of both sealing materials and also on the recalls. Although five areas were scored, for statistical reasons, the worst score among all of the points were selected as it provided the real caries risk condition.

### Caries detection using radiographic analysis

Radiographs were obtained after 1, 2, and 3 years, using the same equipment and settings, and compared to determine the progression of caries during these periods. Radiographic analysis was performed with a viewing box and an x2 magnifying glass, and identified as caries progression. In other words, cases in which the final radiograph showed an increase in any of the directions analyzed (occlusoapical and mesiodistal) detected caries progression. One of five ratings based on the level of confidence of the calibrated operators was scored whether a carious lesion was present in the occlusal surfaces of the teeth^26^:

1 - definitely not caries (sound),

2 - probably not caries,

3 - questionable,

4 - probably caries, and

5 - definitely caries.

### Marginal sealant integrity

The analysis of marginal sealant integrity searched for marginal retentive areas, performed by means of a tactile-visual method (mirror and explorer #5 - DE standard handle, Hu-Friedy, Chicago, IL, USA). The objective of this analysis was to clinically evaluate the retentive areas on probing the marginal surface of the applied sealing material in different directions, suggestive of areas of possible plaque accumulation. In this way, the marginal sealant integrity was clinically evaluated by probing the marginal aspect sealant in four directions: from mesial to distal direction; from distal to mesial direction; from buccal to lingual (palatal) direction; and from lingual (palatal) to buccal direction. Scores were rated using five classifications according to the surface integrity:

Score 0- non retentive;

Score 1- retention in one direction;

Score 2- retention in two directions;

Score 3- retention in three directions;

Score 4- retention in all four directions.

### SEM analysis

After the impressions of all occlusal surfaces, replicas were obtained with epoxy resin (Epoxide, Buehler Ltd, Lake Bluff, IL, USA). The replicas were then mounted on aluminum stubs with a double-sided carbon tape, gold sputter-coated (120 s, 40 mA; SCD 050, JSM-5600LV, Baltec, Vaduz, Liechtenstein) and examined under scanning electron microscopy (SEM; JEOL, Tokyo, TY, Japan), in high vacuum mode and 20 KV of acceleration voltage. Representative images of the changing occlusal aspects of the treated teeth followed up for 3 years were analyzed.

### Call and recalls

Two calibrated operators performed the clinical and radiographic evaluations of the treated teeth in the recalls. Whereas the patients and operators (who determined the allocation sequence) allocated to the intervention group were aware of the determined contralateral tooth that received either one of the sealant treatments, the outcome operators and data analysis were blinded to this information. Blinding was possible as the infiltrant used as a sealing material was not visually distinct from the conventional sealant. After 1, 2, and 3 years of materials placement, selected teeth were submitted again to clinical and radiographic examination. Bitewing radiographies were taken of the area in which the materials were applied. In addition, the occlusal surface integrity was evaluated. In the case of total loss, new sealant was applied to the entire occlusal surface. In the case of total loss, new sealant was applied to the entire occlusal surface. If any signal of caries progression could be perceived (tooth sensitivity, occurrence of visible cavitation or increase in the radiolucent area seen by radiograph) in either group, the tooth would be restored. Regarding the integrity of the sealing material, in the case of partial loss uncovered pits/fissures received a new application of sealant according to the protocol described above.

If any signal of caries progression could be perceived (tooth sensitivity, occurrence of visible cavitation or increase in the radiolucent area seen by radiograph) in either group, the tooth would be restored. Concerning the integrity of the sealing material, in the case of partial loss uncovered pits/fissures, a new application of sealant was performed according to the protocol described above. Caries detection using laser fluorescence method, radiographs, and the marginal integrity method were performed for all occlusal surfaces. Impressions were also taken using a polyvinyl siloxane-based material (Honigum, DMG Dental Materials, Hamburg, Germany) and epoxy replicas were obtained in all of the evaluation periods for scanning electron microscopic analysis (SEM). Three trained professionals organized the data throughout the study.

### Caries progression analysis

An association between the condition of the tooth using the different methods at the end of the study period and experimental groups was also evaluated to determine clinical caries progression. In this study, the experimental groups were followed up at intervals of 1 year over a period of 3 years to allow for interventions in the case of the progression of carious lesions. On each recall, the patients were submitted to anamnesis, and clinical and radiographic examinations were repeated; subjects were asked about the presence of sensitivity to any type of stimuli.

At the end of the observation period, caries progression was defined:

1. Radiographically: when the dimensions of the radiolucent area (in mm) at baseline were compared to those obtained at the other observation periods. Cases in which the final radiograph showed an increase in any of the directions analyzed (occlusoapical and mesiodistal) were defined as caries progression.
2. Using the laser fluorescence method: as previously described, this method gives a numeric value for a lesion. In this way, it is possible to monitor the activity of lesion progression. Changes in the laser fluorescence values correlated positively with the changes in radiographic status.

### Statistical analysis

The sample size of 86 teeth was necessary, considering the possibility of a high dropout rate. According to the estimated outcomes in each group, the type I error level of 0.05, the statistical power (80%) and for continuous outcomes, 86 molars was determined to be the ideal sample size to detect small differences between the two proposed treatments. The software Statistica 8.0 and GraphPad Prism (GraphPad Software, Inc., La Jolla, CA, USA) analyzed the data for each patient in a spreadsheet. Evaluation of intraexaminer agreement by the Kappa test revealed values of .90 for the infiltrant side and .85 for the sealant side. Inter-examiner agreement was 0.82. Descriptive statistical analysis was used. Mann-Whitney determined the caries detection analysis. Friedman test was applied to determine the influence of evaluation times on outcomes. A level of significance of 5% was adopted for all tests. Calculating Spearman’s correlation coefficient analyzed the correlation between the findings of laser fluorescence method and radiographic examination. We also plotted the values measured with laser fluorescence method as functions of the results obtained from radiographic analysis.

## Results

Values obtained from the Cariogram were divided into quintiles, classifying patients as having “rather low” (12%), “low” (6%), “intermediate” (46%), “high” (24%), and “very high” (12%) risk of caries. Only at the 3-year recall, 15 (17.6%, 6 from infiltrant group and 9 from sealant group) of the 86 teeth evaluated (four subjects) were lost to follow-up due to changes in address and/or other reasons (such as iatrogenic pit, fissures restorations, and orthodontic treatment).

Statistical analyses (Spearman Correlation and Mann-Whitney tests) were performed to evaluate the split mouth experimental clinical design to determine whether the side (left or right) at which a material (sealant or infiltrant) was applied would be favored by the initial teeth conditions. Statistics proved that initial conditions had no influence on results and that there was no correlation of application and materials tested.

Table 1 shows the medians of caries detection in function of the material and evaluation times. Statistical analysis using laser fluorescence showed no significance in terms of caries detection, irrespective of evaluation time and sealing materials (p>0.05). In other words, the infiltrant was as effective as the sealant to prevent caries progression.

**Table 1:** Percentual distribution (%) of caries detection using laser fluorescence method

Time	Infiltrant	Sealant
Initial	1 (69.5%); 2 (19.5%); 3 (5.5%); 4 (5.5%)^aA^	1 (69.4%); 2 (16.6%); 3 (8.3%); 4 (5.7%)^aA^
Baseline	1 (91.7%); 2 (5.6%); 3 (2.7%); 4 ( 0%)^aA^	1 (86.1%); 2 (2.8%); 3 (8.3%); 4 (2.8%)^aA^
1 Year	1 (91.7%); 2 (5.6%); 3 (0%); 4 (2.7%)^aA^	1 (86.1%); 2 (8.3%); 3 (2.8%); 4 (2.8%)^aA^
2 Years	1 (94.4%); 2 (0%); 3 (5.6%); 4 (0%)^aA^	1 (97.2%); 2 (2.8%); 3 (0%); 4 (0%)^aA^
3 Years	1 (94.4%); 2 (2.8%); 3 (0%); 4 (2.8%)^aA^	1 (91.6 %); 2 (2.7 %); 3 (0 %); 4 (5.7 %)^aA^

Score 1- from 0 to 13: sound; Score 2- from 14 to 20: enamel cavity; Score 3- from 21 to 29: deep enamel cavities; Score 4- higher than 30: cavity at dentinal level. n=36. Different letters, lower case for columns and upper case for rows: significant (p<0.05)

**Figure 1:**
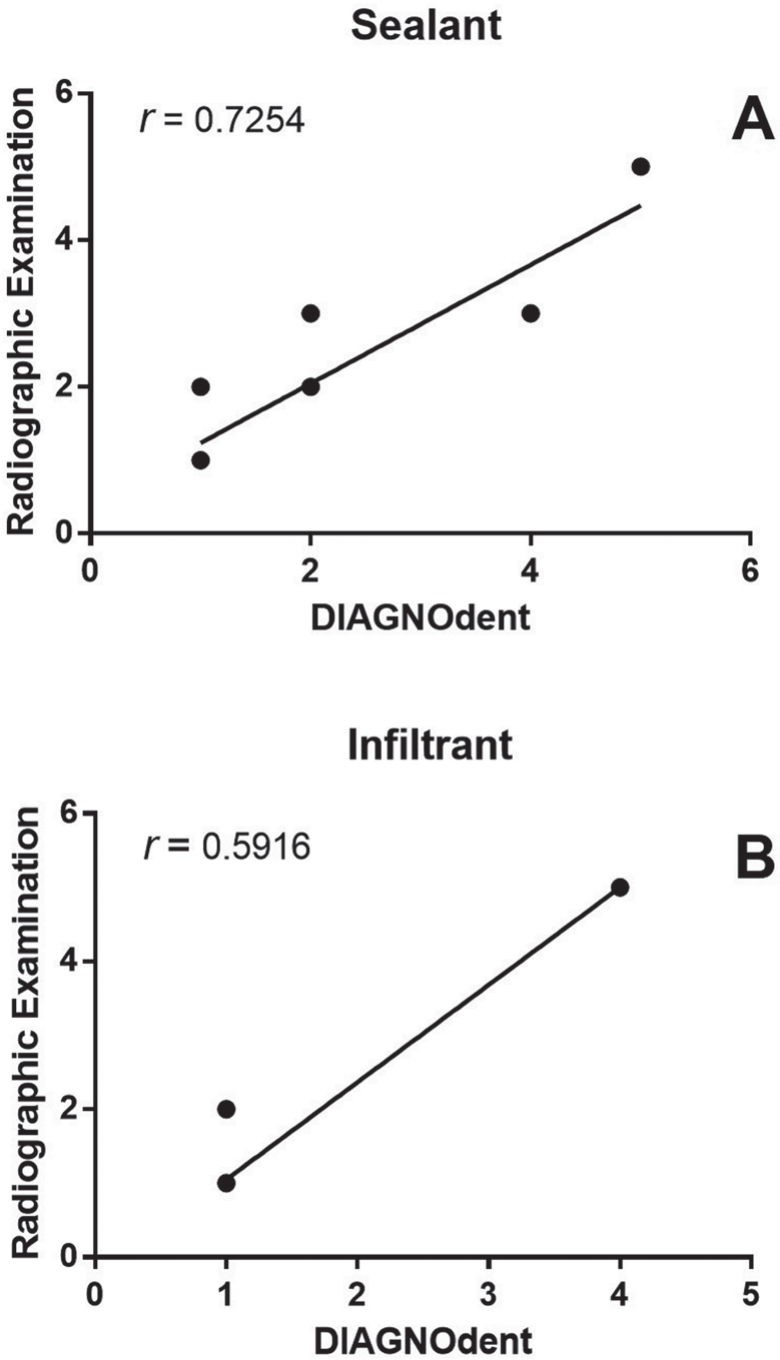
Correlation between the DIAGNOdent method and radiographic analysis for varying sealant material

Table 2 presents the comparative radiographic examination according to sealing material after 3 years. Based on the results, the infiltrant showed significantly better results than that of the sealant (p<0.05). Figure 1 shows the relationships between results of radiography and laser fluorescence, which included two sets of data (sealant and infiltrant). Spearman’s correlation coefficients showed moderate (infiltrant) and strong (sealant) positive correlation between the two methods (Figure 1A and 1B).

**Table 2:** Distribution of caries detection using radiographic analysis after 3 years

Scores	Sealant	Infiltrant
1	27 (67.5)	37 (88.1)
2	8 (20.0)	4 (9.5)
3	4 (10.0)	0 (0.0)
4	0 (0.0)	0 (0.0)
5	1 (2.5)	1 (2.4)
Total	40 (100)	42 (100)
Median	1	1
p-Value	0.0231*	

1=definitely not caries (sound), 2=probably not caries, 3=questionable, 4=probably caries, and 5=definitely caries. *significant (p<0.05)

**Figure 2:**
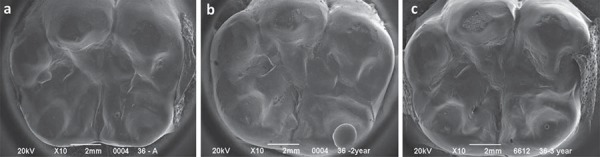
Representative scanning electron microscopy (SEM) micrographs of the infiltrant at (a) the baseline, (b) after 2 years, and (c) 3 years of clinical follow-up. The wear pattern of the material created a uniform surface, which is less favorable to biofilm accumulation

**Figure 3:**
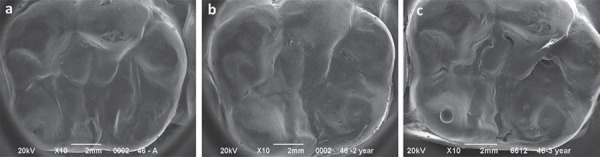
Representative scanning electron microscopy (SEM) micrographs of the sealant at (a) the baseline, (b) after 2 years, and (c) 3 years of clinical follow-up. The wear of the sealant formed an irregular surface, which is more evident after 3 years, favoring biofilm accumulation

Table 3 shows the comparative medians in terms of sealing marginal integrity analysis in function of the material and evaluation time. Statistical analysis comparing the application of both sealing materials was also performed after 1, 2, and 3 years of application. No significance was observed in terms of sealing marginal integrity when the results of both materials were compared, regardless of the evaluation time (p<0.05). Marginal integrity significantly reduced after 1 year for both materials (p<0.05). In spite of this, the marginal adaptation remained stable after 2 and 3 years of evaluation, compared with 1-year results (p>0.05). SEM analysis showed that after 3 years, the infiltrant exhibited a characteristic homogeneous wear pattern (Figure 2). Conversely, the sealant exhibited surface cracking or cratering after the same evaluation time (Figure 3), indicative of retentive areas that favor biofilm accumulation.

**Table 3:** Percentual distribution (%) of sealing marginal integrity

Time	Infiltrant	Sealant
Baseline	0 (100%); 1 (0%); 2 (0%); 3 (0%); 4 (0%)^aA^	0 (100%); 1 (0%); 2 (0%); 3 (0%); 4 (0%^)aA^
1 Year	0 (42.5%); 1 (0%); 2 (18.2%); 3 (3.0%); 4 (36.3%)^bA^	0 (97.0%); 1 (0%); 2 (3.0%); 3 (0%); 4 (0%)^bA^
2 Years	0 (21.2%); 1 (12.1%); 2 (15.2%); 3 (9%); 4 (42.5%)^bA^	0 (30.3%); 1 (6%); 2 (24.3%); 3 (12.2%); 4 (27.2%)^bA^
3 Years	0 (30.3%); 1 (12.1%); 2 (21.3%); 3 (12.1%); 4 (24.2%)^bA^	0 (12.2%); 1 (0%); 2 (9.0%); 3 (6.0%); 4 (72.8%)^bA^

Scores: 0 – non retentive; 1- retention in one direction; 2- retention in two directions; 3- retention in three directions; 4- retention in all four directions. n=36. Different letters, lower case for columns and upper case for rows: significant (p<0.05)

Analysis of clinical caries progression (Table 4) showed no association between the condition of the tooth at the end of the study period and group (P<0.001), with no significance in terms of caries progression when both clinical interventions were compared.

**Table 4:** Absolute and relative frequency of clinical caries progression at the end of the study period

Group	Increase in radiolucent area (Scores 4 and 5)	Laser fluorescence method (Score 4: cavity at dentinal level)
Sealant	1 out of 40 (2.5%)	2 out of 36 (5.6%)
Infiltrant	1 out of 42 (2.4%)	1 out of 38 (2.6%)
p-value	<0.0001*	<0.0001*

*not significant

## Discussion

Pit and fissure sealing has been advocated as the most effective method to prevent occlusal caries progression^28^. In this method, the fissures are isolated from the external cariogenic environment. Numerous preventive and therapeutic treatments in dentistry based on the philosophy of health promotion may somehow interfere with the demineralization of dental tissue by arresting, balancing, or decreasing the progression of carious lesions^19^. Pit and fissure areas are claimed to be eight times more vulnerable than smooth surfaces for dental caries^15^. Thus, the application of an occlusal barrier favored the isolation of the occlusal surface from the surrounding environment, impeditive to the onset of caries.

Dental sealants are indicated as an efficient biofilm control in occlusal areas. Sealants are not resistant to occlusal wear, being partially or completely worn away over time. This may be particularly true for the infiltrant material. It has been advocated that when sealants are partially lost and require repair, the clinician should vigorously attempt to dislodge the remaining sealant material with a probe. If the sealant remains intact to probing, there is no need to completely remove the old material before placing the new^13^. On the other hand, residual sealant parts within the deep occlusal fissure remain to protect this area. In other cases, it is possible that new caries lesions surrounding the sealant margins can occur due to biofilm accumulation. Cases of “biofilm-free” marginal areas certainly contribute for the sealant clinical longevity.

Resin infiltration was primarily developed to arrest proximal lesions^2^. It comprises a methacrylate-based material (89.1% tetraethyleneglycol dimethacrylate) solvated in ethanol (9.9%) with two initiation systems (0.5% camphorquinone and 0.5% ethyl 4-(dimethylamino) benzoate)^17^. Regarding clinical practice, modified etching techniques seem warranted to reduce the influence of the highly mineralized surface layer on infiltration abilities into fissure caries lesions^13^. This clinical trial aimed to evaluate the efficacy of an infiltrant used in pit and fissure sealing as a method to prevent the progression of non-cavitated caries. The research hypothesis, which anticipated that the infiltrant as a sealing material in non-cavitated fissures would not perform as well on caries lesions and sealing marginal integrity as conventional sealant, was rejected. Results were similar in terms of caries detection using laser fluorescence, regardless of evaluation time and sealing materials. Similar results were also observed in terms of sealing marginal integrity when both materials were compared, in despite of the evaluation time. In addition, the infiltrant showed significantly better results after 3 years when bitewing radiographs detected caries.

The diagnosis of the early occlusal lesion, particularly regarding whether the caries was limited to enamel or has involved dentin, is important for differentiating those lesions that are conservatively managed in comparison to those that require restoration. In the latter, the lesions are accurately detected by means of bitewing radiography. Superposing dental tissues and cusps in the anatomical occlusal surface of molars may hamper radiographic analysis in this case. Although the value of bitewing radiography, when used alone, presents relatively low sensitivity in occlusal caries diagnosis, its accuracy is greatly improved when used in conjunction with other techniques. In this way, the use of complementary evaluation methods provides a reliable diagnostic for detecting caries progression.

Caries progression was observed in 1 out of 42 teeth when both materials were applied considering the radiographic analysis (Table 4). Caries progression was also observed in 1 out 38 teeth for the infiltrant and 2 out of 36 teeth when the laser fluorescence method was used. In spite of this difference, no significance was observed among them. The analysis of the results in terms of clinical progression also demonstrated no occurrence of sensitivity or cavitation for both experimental groups. The absence of sensitivity can be explained by the protective barrier provided by both sealing materials, which supports their cariostatic effect on the enamel. These results also showed that the efficacy of this procedure was comparable to that of the sealant to prevent caries progression at the occlusal surface. The similarity in terms of caries progression between the experimental groups evaluated suggests that resin infiltration is suitable to prevent caries progression when applied to sealing occlusal non-cavitated pits and fissures. Thus, it can be stated that the presence of both sealing material on the occlusal surfaces after 3 years of the evaluation can be considered the main reason for this outcome.

A previous study found significantly deeper penetration of the infiltrant into fissure caries lesions by resin infiltration than by a conventional fissure sealant^21^. The authors advocated that the penetration coefficient of the infiltrant was higher than that of a conventional fissure sealant. This coefficient comprises the parameters of viscosity, surface tension, and contact angle of the liquid to the solid^8^. In addition, the infiltrant may also take longer to apply according to the protocols than the sealant. Finally, a more intense erosion of the surface layer, favored by the application of the 15% HCl, may result in deeper penetration of the infiltrant when compared with etching using phosphoric acid gel^7^.

Although both materials compared in this study are methacrylate-based sealers, it seems that the similar results in terms of caries progression, compared with the sealant, are related to facilitated penetrability and improved adhesion of the infiltrant at the pit and fissures intimacy. It has been claimed that the resin infiltration technique was primarily developed to arrest proximal lesions. According with a previous study^17^, compared with the application of sealants, caries infiltration technique allows the diffusion barrier to be shifted from the enamel surface towards the lesion body. In this way, when applying the infiltrant as the micro-invasive treatment of cariogenic lesions in occlusal surfaces, it would be expected that the infiltrant would homogeneously fill up the fissure when compared with the application of a conventional sealant^21^. In this *in vitro* study, this was particularly true for the sealant, which was able to fill most of the fissures completely. For the infiltrant, this was not observed^21^. According to the authors, the penetration of the infiltrant was considered relatively low in the fissure base in comparison with proximal lesions when hydrochloric acid was previously applied.

**Figure 4:**
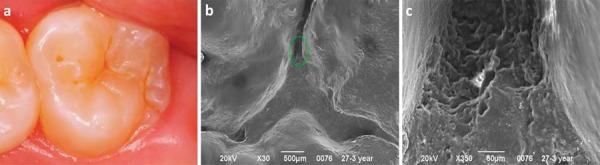
(a) Occlusal aspect of the tooth 27 treated with the infiltrant and clinically followed-up for 3 years; (b) The scanning electron microscopy (SEM) micrograph of the occlusal surface shows a characteristic homogeneous wear pattern, wherein the infiltrate is located below the margins of the oclusal grooves; (c) Higher magnification of the region enclosed by the circle in image b. The infiltrant’s ability to permeate and reach the deepest areas of the occlusal surface is demonstrated. The infiltrant is located in a deeper level in this region, but the tooth remains protected

Clinically, the ability of the infiltrant to penetrate into the fissure may be impaired by the lesion location within the fissure. Also, considering that active and inactive lesions may simultaneously coexist within a fissure, it can be speculated that the ability of the infiltrant to fill up the fissure depends on the wettability of the infiltrant, which is dependent on a sufficient etching. It has been pointed out in an *in vitro* study that the hydrochloric acid may not be able to reach most areas of the fissures^21^. In this way, it was speculated that the lesions are not thoroughly conditioned to allow resin penetration. In addition, in the deepest areas of the fissure there may be entrapped air in occlusal lesions after acid etching and water-rinsing, impeditive for the flow of the infiltrant especially deep into the fissure^21^. These parameters are of particular interest since, in practice, they are not under control of the clinicians. Conversely, in this clinical trial, the microscopic analysis of the sealing material topography revealed a homogeneous wear pattern of the infiltrant throughout the evaluations, different from the more irregular topography observed for the sealant. This was due to a cracked and crated sealant surface, which favors biofilm accumulation (Figures 2 and 3). Although the sealant materials are widely known to present clinically proven efficacy and relatively easy application, not only the retention is claimed to be the main determinant to prevent caries, but also the possibility of their protection remain after partial lost. In many cases, microscopic remaining infiltrant areas appeared at the intimacy of the fissures, demonstrating this material’s ability to permeate and reach the deepest areas of the occlusal surface (Figure 4). This fact can be explained not only by a pre-etching regimen but also to the physical properties of the material, characterized as a low viscosity resin-based infiltrant that utilizes capillary action to penetrate deep into the lesion.

The caries detection method used here and in clinical trial methods such as the laser-diode caries detector has been used in combination with traditional radiography for diagnosis of non-cavitated caries lesions^5^. The effectiveness of laser fluorescence in detecting non-invasive diagnosis of occlusal caries in children was compared to that of visual and radiographic examination^20^. Laser-diode carried detector (high sensitivity) appears useful when associated with visual examination (high specificity) in detecting occlusal caries. The laser fluorescence method is also regarded to present high reproducibility and seems to be more suitable for detecting small superficial lesions^1^. In addition, it has been claimed that sealed occlusal surfaces classified with ICDAS varying from 0 to 4 can be monitored with DIAGNOdent, also helping to predict the need for sealant repairing^10^. In this way, special attention is required when analyzing a condition in which a combination of occlusal surfaces is associated with either a sealant or an infiltrant material. In this study, laser fluorescence caries detection method was able to effectively detect the existence of caries under pit and fissure sealants during a routine recall. Conversely, the laser fluorescence method was not accurate in detecting carious lesions regarding the application of either of the sealing material (Table 1). In addition, the high correlation coefficient proved that laser fluorescence was reliable for quantitatively monitoring carious lesions that reached the dentinal tissue when associated with radiography analysis. Also, in the detection of non-cavitated occlusal caries, there was a strong positive correlation between the results when the laser fluorescence system used with that of the radiographic analysis.

It could be argued that the ICDAS caries detection criteria should be used in the follow-ups. ICDAS clinical diagnostic criteria rely basically on visual assessment and the use of the WHO/PSR probe. Most of soft enamel diagnostic criteria demand the operators to make a subjective clinical judgment based on their experience and knowledge, although previous training and calibrations occur. Even with the use of a detailed system such as the ICDAS, a degree of subjective interpretation is necessary due, perhaps, to visual perception, lighting, and potential bias^12^. Such bias may arise from other surfaces on the same teeth or within other areas of the same surface. The main concern regarding the use of ICDAS scores to evaluate caries progression in the recall relies on the fact that, as a side effect, infiltration treatment was considered to cosmetically camouflage the enamel caries lesions^22^. The masking of enamel caries is caused by infiltrating the lesions using resins with a similar refractive index to that of the apatite crystals^21^. Thus, light scattering is reduced and visual color differences to enamel decreased. In this way, because of the masking of enamel caries due to infiltrant application, the accuracy of visual-based ICDAS scores may be impaired, and this is mainly true for the diagnostic of ICDAS 1 and 2 (visible change in enamel, especially discoloration – white). This somehow explains the reason why no significance was observed when the lesions classified as ICDAS-code 0 and 1 were compared in a previous *in vitro* study^21^.

Concerns have been expressed over the changes in caries pattern and the substantial improvements in the fields of caries diagnosis and caries prevention and treatment. In particular, the use of the dental probe has been continuously criticized as an inappropriate diagnostic tool^14^. The major points of criticism are: the diagnostic outcome is influenced by the dimension of the probe tip and by the subjective probing pressure; the risk of producing irreversible traumatic enamel defects in demineralized occlusal fissures, converting subsurface lesions into cavities favoring lesion progression; the possibility of bacterial contamination from one fissure to another, and the lack of accuracy in the detection of occlusal lesions^14^. Thus, the dental probe has turned out to be almost a “useless” tool in the daily basis clinical practice. Conversely, the great majority of manufacturers generally recommend checking the sealant retention with a probe after polymerization to ensure that all fissures are completely sealed. If not, it should be reapplied, re-etching the exposed fissure. In addition, it has been claimed that an important parameter in the evaluation of the clinical success of sealant materials is marginal adaptation, mainly at the sealant margin^4^. It is true that the traditional methods of evaluating the integrity of the dental sealants, such as visual and probing inspection, cannot identify gaps, adaptation, or failures in the internal structure of sealants. On the other hand, the presence of a marginal gap can lead to marginal staining, which can be considered the first sign of sealant failure^25^. A marginal gap may also imply that there is no occlusal surface isolation against oral microorganisms and, consequently, there is an increased risk for the development of dental caries^16^.

The proposed clinical evaluation of the sealant marginal integrity is not new as has been previously pointed out. In this study, the evaluation of the marginal integrity was “inspired” on ICDAS codes in which each tooth is divided into mesial, distal, facial, lingual, and occlusal surfaces. Considering that this is an *in vivo* study, the use of a probe as a diagnostic tool for the analysis of the marginal integrity of both sealing materials is advantageous because of its simplicity, the easy of recording the data in a presentable form, and the easy of communication among studies. The evaluation of the marginal areas surrounding the sealing materials is extremely important as these would be areas of biofilm stagnation, thereby increasing the risk for development of dental caries next to fissure-sealed areas and complementing the results.

Possible limitations of this study are the sample size of 86 teeth, the sample lost, and the period of evaluation of the experimental groups. Conversely, the marked difference in terms of caries progression observed between the two groups indicates a satisfactory sample size for the analysis of this intervention and an appropriate observation period of three years for the detection of differences considering the distinct methods of analysis. In addition, based on the findings of the present study, it can be inferred that the drop-outs were not able to affect the results.

Briefly, the results from this study suggest that the infiltrant is effective at arresting occlusal caries progression compared with a conventional sealant. Although marginal integrity significantly reduced after 1 year, this non-invasive treatment seems to protect the tooth structure as similar retentive areas were found at 3-year evaluation time. This helps avoid the repetitive restorative cycle that would otherwise increase the risks of adverse effects on the remaining tooth structure. In addition, radiographic analysis after 3 years showed significantly better results when the infiltrant was applied to non-cavitated lesions compared to that of the conventional sealant. In this way, this study proved the clinical effect of the deeper penetration of the infiltrant on the inhibition of lesion progression. This non-invasive treatment minimizes the possibility of secondary caries and maintains the longevity of the dentition for a prolonged period, thereby emphasizing its importance.

## Conclusions

With the criteria used to evaluate the clinical performance of these materials, it can be concluded that:

Similar results in terms of marginal sealant integrity were observed after 3 years;

An explorer-probe of the infiltrant presents more regular wear after 3 years of clinical application;

Less caries progression occurs after 3 years when the infiltrant is applied on non-cavitated fissures in the radiographic analysis;

Resin infiltration seems to be suitable to prevent caries progression when applied to sealing occlusal non-cavitated pits and fissures.
